# Safety and feasibility of D3 lymph node dissection in oldest-old patients undergoing colorectal cancer surgery: a multi-institutional, retrospective analysis

**DOI:** 10.1007/s10151-025-03187-3

**Published:** 2025-07-19

**Authors:** R. Inada, F. Teraishi, T. Mitsuhashi, S. Takanaga, T. Toshima, T. Ohtani, R. Yoshida, N. Hori, K. Shigemitsu, S. Yamamoto, T. Kubota, Y. Okano, T. Nobuhisa, F. Taniguchi, W. Ishikawa, R. Shoji, T. Matsuda, T. Umeoka, T. Fujiwara, Setouchi Colorectal Neoplasm Registration Study Group Collaborators

**Affiliations:** 1 Department of Surgery, Kochi Health Sciences Center, Kochi, Japan; 2 Department of Gastroenterological Surgery, Okayama University Graduate School of Medicine, Dentistry and Pharmaceutical Sciences, Okayama, 700-8558 Japan; 3 Center for Innovative Clinical Medicine, Okayama University Hospital, Okayama, Japan; 4 Department of Surgery, Kagawa Rosai Hospital, Marugame, Japan; 5 Department of Surgery, Saiseikai Okayama Hospital, Okayama, Japan; 6 Department of Surgery, Okayama Rosai Hospital, Okayama, Japan; 7 Department of Surgery, Tottori Municipal Hospital, Tottori, Japan; 8 Department of Surgery, Tsuyama Chuo Hospital, Tsuyama, Japan; 9 Department of Surgery, Okayama City Hospital, Okayama, Japan; 10 Department of Surgery, Kobe Red Cross Hospital, Kobe, Japan; 11 Department of Surgery, Onomichi City Hospital, Onomichi, Japan; 12 Department of Surgery, Himeji Red Cross Hospital, Himeji, Japan; 13 Department of Surgery, National Hospital Organization Iwakuni Clinical Center, Iwakuni, Japan; 14 Department of Surgery, Fukuyama City Hospital, Fukuyama, Japan; 15 Department of Surgery, Matsuda Hospital, Kurashiki, Japan; 16 Department of Surgery, Matsuyama City Hospital, Matsuyama, Japan

**Keywords:** Lymph node dissection, Colorectal cancer, Oldest-old patients, Postoperative complication

## Abstract

**Background:**

Colorectal cancer (CRC) is a significant health burden, with lymph node dissection (LND) playing a critical role in staging and guiding treatment. However, the optimal extent of LND for the oldest-old population (aged ≥ 90 years) remains undefined because of insufficient targeted clinical data. This study aimed to compare the short-term outcomes of D3 versus non-D3 LND in Stage II–III CRC in oldest-old patients.

**Methods:**

This retrospective cohort study utilized data from the Setouchi Colorectal Neoplasm Registration database, including 282 oldest-old patients with CRC treated between 2011 and 2022. Patients were stratified into D3 and non-D3 LND groups, with inverse-probability-weighted regression adjustment implemented to address potential confounding factors. Postoperative complications and hospital stays were analyzed using regression models and descriptive statistics.

**Results:**

D3 LND resulted in significantly higher lymph node harvests in both Stage II and Stage III patients (*p* < 0.01). There were no significant differences in overall or major postoperative complications between D3 and non-D3 groups. Hospital stays were comparable for Stage II patients but shorter for Stage III patients in the D3 group (*p* < 0.01). Complication rates ranged from 28% to 47.7%, with surgical site infections and pneumonia being the most common.

**Conclusions:**

D3 LND can be safely performed in oldest-old patients with CRC without increasing postoperative complications or extending hospital stays. These findings support the feasibility of extensive LND in this age group, but further studies are needed to evaluate its oncological benefits.

**Supplementary Information:**

The online version contains supplementary material available at 10.1007/s10151-025-03187-3.

## Introduction

Colorectal cancer (CRC) is one of the most commonly diagnosed malignancies worldwide [1] and represents a significant public health burden in terms of both morbidity and mortality. The standard of care for CRC is a multimodal approach including surgery, chemotherapy, and, in some cases, radiotherapy. Surgical resection remains the cornerstone of curative treatment. One of the critical components of oncologic surgery for CRC is the thorough evaluation of lymph nodes, which plays a pivotal role in staging, prognosis, and guiding adjuvant therapy decisions.

The lymphatic system serves as a major pathway for the spread of CRC, and accurate pathological assessment of lymph nodes is critical for the identification of metastatic disease. The Union for International Cancer Control (UICC) and the American Joint Committee on Cancer (AJCC) have long emphasized the importance of lymph node assessment in the TNM staging system [2]. In this system, the “N” component specifically reflects the number of lymph nodes involved by metastatic disease, which is directly correlated with survival outcomes and risk of recurrence. Therefore, it is important to ensure that an adequate number of lymph nodes are removed and examined to avoid understaging, which could lead to suboptimal treatment.

In colorectal cancer surgery, central vascular ligation, complete mesocolic excision for colon cancer, and total mesorectal excision for rectal cancer are the standard surgical methods in Western countries [3, 4]. In Japan, the extent of lymph node dissection (LND) is based on the stage of colorectal cancer, with treatment guidelines recommending D3 dissection for Stage II and Stage III [5]. Both techniques are based on similar principles and do provide good oncological outcomes. Surgeons must adhere to these principles to optimize LND in CRC resection. However, there is a lack of high-quality data to guide appropriate LND for oldest-old patients, since many clinical studies have often involved younger, healthier patients. Regarding LND in older patients with colorectal cancer, several reports have examined colorectal cancer in patients aged 70 or 75 years and older. While standard D3 LND can be safely performed, findings on prognosis remain inconsistent. Some studies report survival benefits, whereas others indicate that D3 LND in older patients does not demonstrate prognostic improvement [6–9]. The aim of this study was to investigate the appropriate extent of LND in surgery for Stage II–III colorectal cancer in the oldest-old patients by comparing the short-term outcomes of patients with non-D3 and those with D3 dissections.

## Materials and methods

### Patients

**Fig. 1:**
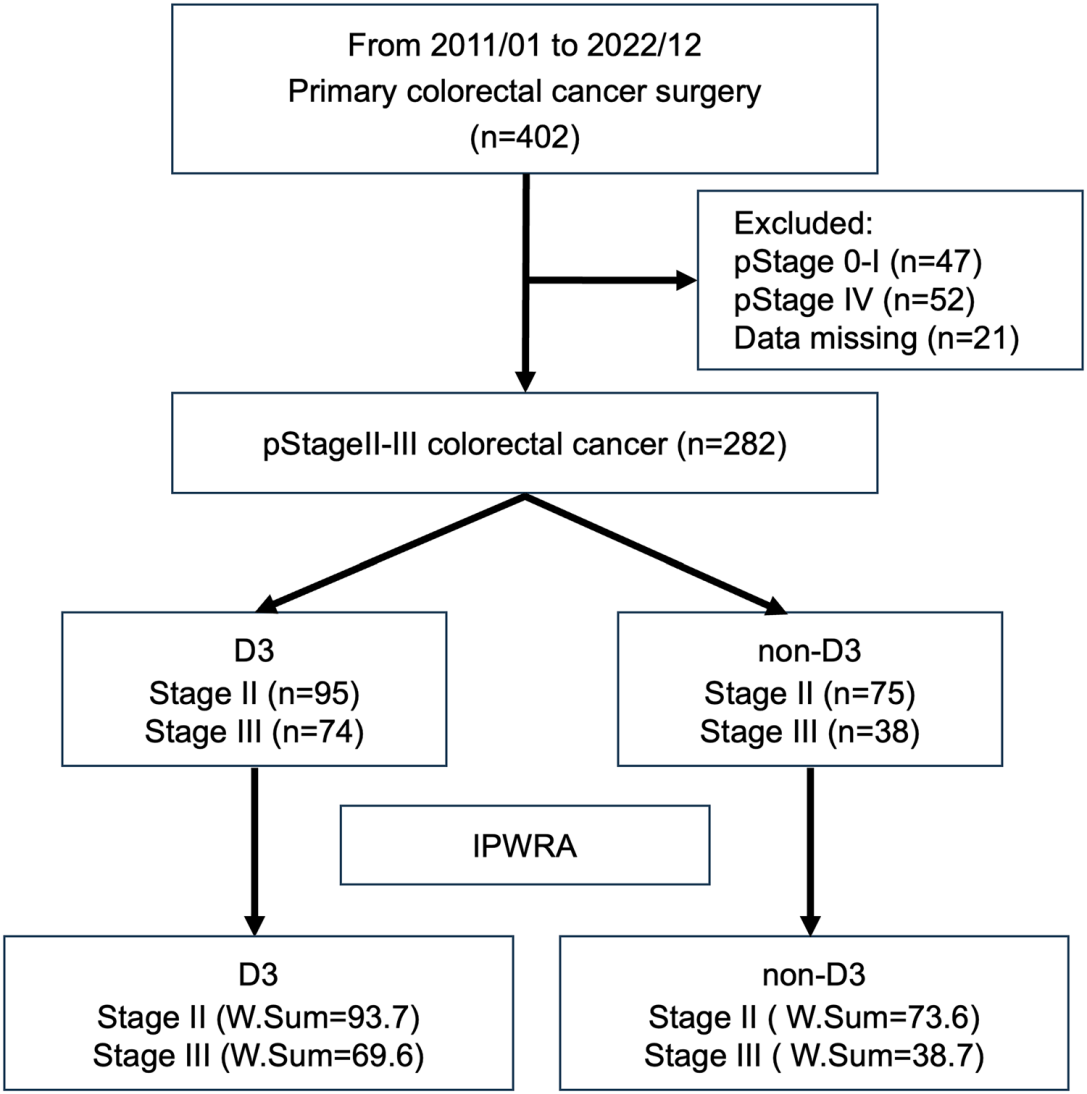
Flowchart of patient selection. *IPWRA* inverse-probability-weighted regression adjustment, *pStage* pathological stage, *W.Sum* weighted sum

The ethics committee of Okayama University Hospital approved this retrospective study (approval number 2112-036) and all participating hospitals approved this study as exempt human subject research. Study data were collected and managed using Research Electronic Data Capture (REDCap) tools hosted at Okayama University Hospital. This retrospective, cohort study used data from the Setouchi Colorectal Neoplasm Registration study database, which collected information on oldest-old patients aged 90 years or older from 15 Okayama University-affiliated hospitals between January 2011 and December 2022. A total of 403 cases of colorectal cancer in oldest-old patients were identified. The inclusion criteria were patients with colorectal cancer undergoing non-D3 or D3 LND for pathological Stage II or III cancer, and exclusion criteria included Stage 0–I cancer, Stage IV cancer, and cases with missing data. Screening results showed that 170 Stage II and 112 Stage III patients were included in the study sample (Fig. 1).

### Research Electronic Data Capture (REDCap)

REDCap is a secure, web-based software platform designed to support data capture for research studies, providing (1) an intuitive interface for validated data capture; (2) audit trails for tracking data manipulation and export procedures; (3) automated export procedures for seamless data downloads to common statistical packages; and (4) procedures for data integration and interoperability with external sources [10, 11].

### Definition of lymph node stations and D3 LND

The definition of lymph node (LN) stations was based on the Japanese Classification of Colorectal, Appendiceal, and Anal Carcinoma. LN stations are defined as follows: Intermediate LNs are located along the ileocecal, right colonic, middle colonic, left colonic, sigmoid, and inferior mesenteric arteries, between the origin of the artery and the terminal colonic artery. Main LNs are located at the origin of the ileocecal, right colonic, middle colonic, and inferior mesenteric arteries (Supplementary Figure). D3 dissection was defined as resection of the para-intestinal, intermediate, and main lymph nodes [12]. The selection of surgical procedures, including LND, was determined through preoperative conferences at each participating institution.

### Outcome measurement

Incidence of postoperative complications was evaluated according to the Clavien–Dindo classification [13, 14]. Any deviation from normal postoperative status was considered a complication, and the treatment required to correct the complication was the basis for the grade of severity. Thus, complications that required medical therapy were classified as Grade II; complications that required surgical, endoscopic, or radiologic treatment as Grade III; life-threatening complications as Grade IV; and postoperative death as Grade V. In this study, Grade II or higher were considered as having postoperative complications. Complications that occurred in the first 30 days after surgery were registered by the physician in charge. The primary outcome was the incidence of all postoperative complications according to the Clavien–Dindo classification. 

### Statistical analysis

Statistical analysis of parametric variables was expressed as median (interquartile range), and categorical variables were expressed as number (%). Categorical variables were compared between the non-D3 and D3 groups using Fisher’s exact test or chi-square test, while continuous variables were compared using the Mann–Whitney *U* test.

For postoperative complications, differences between non-D3 and D3 groups were calculated as risk ratios and their 95% confidence intervals (CIs) using modified Poisson regression. 95% CIs were calculated using the bootstrap method. Inverse-probability-weighted regression adjustment (IPWRA), which is doubly robust, was employed to adjust for confounding factors [15]. In the IPWRA treatment model (i.e., inverse probability weighting), propensity scores were calculated using a logistic regression model, with age, gender, BMI, ASA classification, and surgical approach as explanatory variables and surgical approach (non-D3 or D3) as the response variable. These propensity scores were used to calculate the stabilized weights in the IPWRA method using the following formula:

In the non-D3 group,

$$\text{weight}=\frac{\text{Pr(non D3 dissection)}}{\text{propensity score}}$$

In the D3 group,

$$\text{weight}=\frac{\text{Pr(D3 dissection)}}{1-{\text{propensity score}}}$$

In the IPWRA outcome model (i.e., regression adjustment), a modified Poisson regression was performed with the same variables to calculate the propensity score and estimate the risk of postoperative complications.

All statistical analyses were conducted using Stata/MP (version 18.0, Stata Corp, College Station, TX, USA). *P* < 0.05 was considered statistically significant. Because this study has an exploratory component, a multiplicity of tests was not considered.

## Results

The clinical characteristics of 169 D3 lymph node dissection (LND) and 113 non-D3 LND patients are shown in Table 1. In D3 LND there were 117 female patients (69.2%) and 52 male patients (30.8%), while in non-D3 LND there were 69 female patients (61.1%) and 44 male patients (38.9%). The body mass index (BMI) was 20.5 kg/m^2^ in the D3 LND group and 20.0 kg/m^2^ in the non-D3 group. Ninety-seven patients (57.4%) in D3 LND and 66 patients (58.4%) in non-D3 LND had an ASA classification of 3 or higher. Among D3 LND patients, 137 had colon cancer and 32 had rectal cancer, with right-sided colon cancer (from the cecum to the transverse colon) accounting for 106 cases (62.7%). In non-D3 LND, 89 patients had colon cancer and 24 had rectal cancer, with right-sided colon cancer present in 59 cases (52.2%). D3 LND was performed in 85 cases (50.3%) of clinical stage (cStage) II and 69 cases (40.8%) of cStage III, while non-D3 LND was performed in 73 cases (64.6%) of cStage II and 31 cases (27.4%) of cStage III. Regarding the surgical approach, D3 LND patients underwent open surgery in 62 cases (36.7%) and laparoscopic surgery in 107 cases (63.3%), while non-D3 LND patients underwent open surgery in 82 cases (72.6%) and laparoscopic surgery in 31 cases (27.4%). Two patients with rectal cancer were treated with preoperative radiotherapy (data not shown).

**Table 1:** Baseline patient characteristics before inverse-probability-weighted regression adjustment (IPWRA)

	All (*n* = 282)	D3 (*n* = 169)	Non-D3 (*n* = 113)
Age, years, median (IQR)	92 (91–94)	92 (91–94)	93 (91–95)
Gender, *n* (%)			
Male	96 (34.0)	52 (30.8)	44 (38.9)
Female	186 (66.0)	117 (69.2)	69 (61.1)
BMI, kg/m^2^, median (IQR)	20.2 (18.1–22.4)	20.5 (18.1–22.5)	20.0 (18.1–22.2)
ASA classification, *n* (%)			
2	116 (41.1)	71 (42.0)	45 (39.8)
3	159 (56.4)	95 (56.2)	64 (56.6)
4	4 (1.4)	2 (1.2)	2 (1.8)
Unknown	3 (1.1)	1 (0.6)	2 (1.8)
Tumor location, *n* (%)			
Right	165 (58.5)	106 (62.7)	59 (52.2)
Cecum	39 (13.8)	29 (17.1)	10 (8.8)
Ascending colon	92 (32.6)	63 (37.3)	29 (25.8)
Transverse colon	34 (12.1)	14 (8.3)	20 (17.6)
Left	61 (21.6)	31 (18.3)	30 (26.4)
Descending colon	12 (4.3)	2 (1.2)	10 (8.8)
Sigmoid colon	49 (17.3)	29 (17.1)	20 (17.6)
Rectum	56 (19.9)	32 (19.0)	24 (21.4)
cStage, *n* (%)			
I	18 (6.4)	12 (7.1)	6 (5.3)
II	158 (56.0)	85 (50.3)	73 (64.6)
III	100 (35.5)	69 (40.8)	31 (27.4)
Unknown	6 (2.1)	3 (1.8)	3 (2.7)
Surgical approach, *n* (%)			
Open	144 (51.1)	62 (36.7)	82 (72.6)
Laparoscopy	138 (48.9)	107 (63.3)	31 (27.4)

*ASA* American Society of Anesthesiologists, *BMI* body mass index, *IQR* interquartile range, *pStage* pathological stage

Surgical outcomes are shown in Table 2. In pStage II cases, there were significantly more emergency surgery cases in the non-D3 group. Operative time was significantly longer in the D3 group compared to the non-D3 group for Stage II patients (210 min vs. 170 min, *p* < 0.01). No significant difference was observed in Stage III patients. Blood loss was similar between groups. The number of lymph nodes harvested was significantly higher in the D3 group for both stages (Stage II: 19 vs. 11 nodes; Stage III: 17 vs. 12 nodes; *p* < 0.01 for both comparisons). ICU admission rates did not differ significantly between groups. Regarding postoperative complications, the overall incidence (all Clavien–Dindo grades) in Stage II was 33 cases (38.4%) in the D3 group and 32 cases (42.7%) in the non-D3 group, while in Stage III it was 21 cases (28.4%) in the D3 group and 18 cases (47.7%) in the non-D3 group, with no significant differences observed. Major complications (Clavien–Dindo grade III or higher) occurred in 5 cases (5.3%) of the D3 group and 6 cases (8%) of the non-D3 group in Stage II, and in 3 cases (4.1%) of the D3 group and 5 cases (13.2%) of the non-D3 group in Stage III, with no significant differences. Three deaths occurred within 30 days in Stage II patients, all due to pneumonia-related respiratory complications. Postoperative hospital stay was 15 days in the D3 group versus 17 days in the non-D3 group for Stage II (*p* = 0.07) and 14 days versus 18 days for Stage III, with significantly longer stays in the non-D3 group (*p* < 0.01). One reoperation was required in the non-D3 group of Stage III patients because of wound dehiscence. Details of major postoperative complications are presented in Supplementary Table 1. Surgical site infection was the most common complication, occurring in 2 and 9 cases in the Stage II D3 and non-D3 groups, respectively, and in 2 and 5 cases in the Stage III D3 and non-D3 groups, respectively. Pneumonia was the second most common complication, occurring in 3 cases in the Stage II D3 group, 9 cases in the Stage II non-D3 group, and 4 cases in the Stage III D3 group. Urinary tract infections occurred in 4 cases in the Stage II D3 group, 1 case in the Stage II non-D3 group, 5 cases in the Stage III D3 group, and 3 cases in the Stage III non-D3 group.

**Table 2:** Surgical outcomes of patients before IPWRA

	pStage II (*n* = 170)				pStage III (*n* = 112)			
	All	D3	Non-D3	*p* value	All	D3	Non-D3	*p* value
	*N* = 170	*N* = 95	*N* = 75		*N* = 112	*N* = 74	*N* = 38	
Tumor location, *n* (%)								
Right	103 (60.6)	64 (67.4)	39 (52.0)	0.05	62 (55.4)	42 (56.8)	20 (52.6)	0.57
Left	36 (21.2)	14 (14.7)	22 (29.3)		24 (21.4)	17 (23.0)	7 (18.5)	
Rectum	30 (17.6)	17 (17.9)	13 (17.3)		26 (23.2)	15 (20.2)	11 (28.9)	
Emergency operation, *n* (%)	17 (10.0)	5 (5.3)	12 (16.0)	0.02*	6 (5.4)	4 (5.4)	2 (5.3)	0.97
Operation time, min, median (IQR)	191 (135–251)	210 (145–273)	170 (121–224)	< 0.01*	196 (140–251)	203 (148–253)	184 (119–232)	0.09
Blood loss, ml, median (IQR)	30 (10–115)	30 (10–70)	50 (5–155)	0.30	50 (10–108)	30 (10–100)	50 (10–200)	0.30
Lymph node yield, median (IQR)	15 (9–23)	19 (13–26)	11 (5–16)	< 0.01*	15 (10–24)	17 (12–27)	12 (7–18)	< 0.01*
ICU admission, *n* (%)	48 (28.2)	22 (23.2)	26 (34.7)	0.10	30	18 (24.3)	12 (31.6)	0.41
Postoperative complications								
C–D Grade I–V, *n* (%)	65 (38.2)	33 (38.4)	32 (42.7)	0.29	39	21 (28.4)	18 (47.4)	0.05*
C–D Grade III–V, *n* (%)	11 (6.5)	5 (5.3)	6 (8)	0.47	8	3 (4.1)	5 (13.2)	0.08
30-day mortality, *n* (%)	3 (1.8)	1 (1.1)	2 (2.7)		0 (0)	0 (0)	0 (0)	
Length of stay, days, median (IQR)	16 (12–23)	15 (11–21)	17 (12–25)	0.07	16 (11–23)	14 (11–21)	18 (14–28)	0.01*
Reoperation, *n* (%)	0 (0)	0 (0)	0 (0)		1 (0.9)	0 (0)	1 (2.6)	

*C−D* Clavien–Dindo classification, *IQR* interquartile range, *pStage* pathological stage

*Statistically significant

Table 3 shows the clinical characteristics after IPWRA, showing an improved balance between the D3 and non-D3 groups in both Stage II and Stage III patients.

**Table 3:** Clinical characteristics of patients after IPWRA

	pStage II							
	Unweighted				Weighted			
	D3	Non-D3	Balance		D3	Non-D3	Balance	
	*N *= 95	*N* = 75	Std-diff	Var-ratio	W.Sum = 93.7	W.Sum = 73.6	Std-diff	Var-ratio
Age, years, mean (SD)	92.0 (2.0)	92.0 (4.0)	0.305	1.623	92.0 (3.0)	92.0 (3.0)	0.000	1.202
BMI, kg/m^2^, mean (SD)	20.6 (4.2)	20.0 (4.6)	0.140	1.098	20.3 (4.4)	20.2 (5.5)	0.007	1.111
Gender								
Male	31 (32.6%)	31 (41.3%)			34.5 (36.9%)	25.9 (35.2%)		
Female	64 (67.4%)	44 (58.7%)	0.195	1.109	59.1 (63.1%)	47.7 (64.8%)	0.035	0.983
ASA classification								
2	39 (41.1%)	31 (41.3%)			39.0 (41.6%)	31.4 (42.7%)		
3	55 (57.9%)	43 (57.3%)	0.008	1.000	53.9 (57.6%)	41.6 (56.5%)	0.022	1.009
4	1 (1.1%)	1 (1.3%)	0.028	1.288	0.8 (0.8%)	0.6 (0.8%)	0.001	0.992
Surgical approach								
Open	36 (37.9%)	53 (70.7%)			49.2 (52.5%)	38.3 (52.1%)		
Laparoscopy	59 (62.1%)	22 (29.3%)	0.697	0.870	44.5 (47.5%)	35.3 (47.9%)	0.009	1.004
Balance average			0.229	1.165			0.012	1.050

	pStage III							
	Unweighted				Weighted			
	D3	non-D3	Balance		D3	non-D3	Balance	
	*N* = 74	*N* = 38	Std-diff	Var-ratio	W.Sum = 69.6	W.Sum = 38.7	Std-diff	Var-ratio
Age, years, mean (SD)	92.0 (3.0)	93.0 (3.0)	0.203	2.041	92.0 (3.0)	92.0 (3.0)	0.070	1.857
BMI, kg/m^2^, mean (SD)	20.2 (4.4)	20.1 (2.6)	0.041	0.717	20.2 (4.3)	20.4 (2.3)	0.109	0.664
Gender								
Male	21 (28.4%)	12 (31.6%)			21.9 (31.4%)	12.7 (32.8%)		
Female	53 (71.6%)	26 (68.4%)	0.065	1.072	47.8 (68.6%)	26.0 (67.2%)	0.031	1.037
ASA classification								
2	32 (43.8%)	13 (34.2%)			28.4 (40.8%)	15.8 (40.8%)		
3	40 (54.8%)	24 (63.2%)	0.179	0.949	40.3 (57.8%)	22.4 (57.9%)	0.001	1.012
4	1 (1.4%)	1 (2.6%)	0.084	1.842	0.9 (1.4%)	0.5 (1.3%)	0.003	0.989
Surgical approach								
Open	26 (35.1%)	29 (76.3%)			34.0 (48.8%)	18.5 (47.8%)		
Laparoscopy	48 (64.9%)	9 (23.7%)	0.924	0.812	35.6 (51.2%)	20.2 (52.2%)	0.021	1.011
Balance average			0.249	1.239			0.039	1.095

*SD* standard deviation, *Std-diff* standardized mean difference, *Var-ratio* variance ratio, *W.Sum* weighted sum

As shown in the upper panel of Table 4, the adjusted risk ratios for postoperative complications in the non-D3 group compared to the D3 group were 1.24 (95% CI 0.80–1.94, *p* = 0.335) for Stage II and 1.62 (95% CI 0.62–4.22, *p* = 0.321) for Stage III, both of which were not statistically significant. The lower panel of Table 4 shows the adjusted risk ratios for postoperative hospital stay in the non-D3 group compared to the D3 group: 1.19 (95% CI 0.80–1.77, *p* = 0.385) for Stage II and 1.09 (95% CI 0.71–1.66, *p* = 0.698) for Stage III, with no statistical significance observed.

**Table 4:** Risk ratio of postoperative complications for non-D3 LND based on D3 LND (upper panel). Length of hospital stay ratio for non-D3 LND based on D3 LND (negative binomial regression analysis, lower panel)

	Number	Risk ratio (95% CI)	*p* value
Stage II			
Crude model	170	1.23 (0.84, 1.80)	0.291
Adjusted model	167	1.24 (0.80, 1.94)	0.335
Stage III			
Crude model	112	1.67 (1.02, 2.74)	0.043
Adjusted model	108	1.62 (0.62, 4.22)	0.321

	Number	Day ratio (95% CI)	*p* value
Stage II			
Crude model	160	1.23 (0.71, 2.13)	0.463
Adjusted model	158	1.19 (0.80, 1.77)	0.385
Stage III			
Crude model	107	1.34 (0.98, 1.82)	0.066
Adjusted model	103	1.09 (0.71, 1.66)	0.698

## Discussion

The results of this study showed that D3 LND in oldest-old patients with colorectal cancer can be performed safely without increased postoperative complications than in non-D3 LND cases. Moreover, postoperative hospital stay was not prolonged in the D3 LND group. The strengths of this study include the large number of cases collected: previous reports of surgical outcomes in patients with colorectal cancer aged 90 years or older typically involved around 100 cases [16–18], and this is the first report using IPWRA to discuss optimal LND in a large number of oldest-old patients with colorectal cancer.

The incidence of CRC increases with age, with a considerable number of cases diagnosed in individuals over 90 years [1]. While advancements in surgical techniques and perioperative care have improved outcomes for older patients in general, nonagenarians represent a unique subgroup with higher rates of comorbidities and frailty, factors that significantly influence treatment decisions [19, 20]. The initial challenge lies in determining the suitability for surgical intervention, as non-operative management is also an option [21]. While surgery offers the prospect of improved survival in selected patients [18, 22, 23], the risk of postoperative complications and mortality is considerably high [16].

In nonagenarian patients, the benefits of extensive LND are less clear. Evidence suggests that the rate of lymph node metastasis may decrease with age, and tumor biology may differ in older patients [24]. This could imply that the benefit from a more radical D3 dissection might not be as substantial as in younger individuals. Indeed, a Japanese propensity score-matched study found survival benefit of LND in elderly patients, but did not specifically focus on the efficacy of D3 dissection in nonagenarians [25]. Furthermore, a multicenter study by Lu et al., the RELARC trial, randomized patients with right-sided colon cancer to either D2 or D3 lymphadenectomy and found no significant difference in disease-free survival or overall survival [26]. This questions the routine use of D3 dissection, even in the younger population.

On the other hand, several studies on older patients indicate that survival benefit can be achieved in those who undergo resection with adequate lymph node retrieval [18, 22, 23, 25]. However, these are largely observational studies which lack a comparison group receiving limited nodal clearance. Thus, the exact extent of LND remains ambiguous for the frail, elderly patient. This further emphasizes the need for individual patient assessment and tailoring treatment plans.

The incidence of postoperative complications following colorectal cancer surgery is higher in older patients than in younger patients, ranging from 25.9% to 66.2%, as reported by various authors [17, 18, 22, 23]. In the present study, the complication rate ranged from 28% to 47.4%, similar to previous reports. The risk of adverse surgical outcomes in nonagenarians is influenced by several patient-specific factors, including age, comorbidities, frailty, and nutritional status [19, 20, 27, 28]. Kim and Kim identified factors associated with postoperative complications and 1-year mortality in octogenarians and nonagenarians, emphasizing the importance of careful patient selection [20]. Frailty, in particular, has emerged as a significant predictor of poor outcomes following surgery. In the database used for this study, frailty scores other than ASA-PS were not collected, and it remains unclear whether these would have influenced the selection of surgical procedures. Prehabilitation programs, involving exercise and nutritional support, may improve perioperative outcomes in frail older patients [29]. In this study, there was no standardized protocol for perioperative management, suggesting potential inter-institutional variations in postoperative care.

The fact that D3 dissection did not lead to an increase in postoperative complications compared with non-D3 dissection may be due to selection bias, because surgeons chose non-D3 dissection at their discretion based on patient risk. In the present study, the ASA classification for preoperative risk assessment was matched using IPWRA, ensuring that the overall health status of both groups was equivalent and minimizing selection bias. Minimally invasive surgical techniques, particularly laparoscopy, have gained popularity in CRC surgery, showing potential advantages in older patients [30–32]. Studies indicate that laparoscopic surgery is associated with lower postoperative morbidity, shorter hospital stays, and earlier return to normal function [32–36]. A multicenter study of older patients, by Rinaldi et al., suggests that in selected patients over the age of 80, laparoscopy may offer improved outcomes compared to open procedures [32]. This is especially relevant for nonagenarians, who are more likely to experience significant morbidity from major open surgery. However, these studies did not analyze the extent of LND performed using laparoscopic approaches. In this study, since both open and laparoscopic surgical approaches were matched using IPWRA, it is considered that the short-term outcomes between D3 and non-D3 LND were not influenced by the surgical approach. 

There are several limitations to this study. First, it was a retrospective study. However, no multicenter, cohort study of colorectal cancer surgery patients aged 90 years or older has examined such a large number of patients as the present study, and the background characteristics were to some extent matched by IPWRA, making the study results regarding optimal LND in oldest-old patients undergoing colorectal cancer surgery notable. Second, there was inter-institutional bias. It would be difficult to standardize surgical principles among the participating centers in the present study, because some centers perform laparoscopic surgery aggressively, whereas others prefer open surgery as the first choice. Furthermore, as the study did not restrict the operating surgeons, there may be interoperator bias present in our findings. The JCOG 0404 study also showed some differences in the long-term results of laparoscopic surgery, but there were certain inter-institutional differences [37]. Third, it should be noted that this study did not examine the association between LND and prognosis. Although we are currently in the process of collecting prognostic data, the effort has been challenging. While short-term follow-up has been adequately conducted at most institutions, there are very few cases with outpatient follow-up exceeding 1 year. This limitation is primarily due to the difficulty older patients face in adhering to regular follow-up schedules, as well as their frequent relocation to care facilities. As a result, the availability and completeness of follow-up data are often restricted.

In conclusion, LND remains a cornerstone in the surgical management of colorectal cancer. On the basis of the findings of this study, D3 LND for Stage II and III colorectal cancer in oldest-old patients appears to be safely performed without increasing postoperative complications, provided there are no issues with preoperative assessment of surgical tolerance. Moving forward, further investigations are needed to address the critical clinical question of whether D3 LND improves oncological outcomes in oldest-old patients with colorectal cancer.

## Supplementary Information

Below is the link to the electronic supplementary material.

Supplementary file1 (DOCX 156 KB)

Supplementary file2 (PDF 11 KB)

## Data Availability

The data that support the findings of this study are available from the corresponding author upon reasonable request. No datasets were generated or analysed during the current study.
