# Supplementary material for: Safety and feasibility of D3 lymph node dissection in oldest-old patients undergoing colorectal cancer surgery: a multi-institutional, retrospective analysis
Source: Tech Coloproctol. 2025 Jul 19;29(1):146. doi: 10.1007/s10151-025-03187-3 (PMC12276142; doi:10.1007/s10151-025-03187-3)

**Supplementary Figure** Lymph node groups and station numbers

Red: Pericolic/perirectal lymph nodes

Blue: Intermediate lymph nodes

Yellow: Main lymph nodes

Green: Lateral lymph nodes

Gray: Downward lymph nodes

White: Lymph nodes proximal to the main lymph nodes

Reprinted with permission from the Japanese Society for the Cancer of the Colon and Rectum and Kanehara & Co. Appendiceal, and

Anal Carcinoma- the 3rd English edition, 2019.


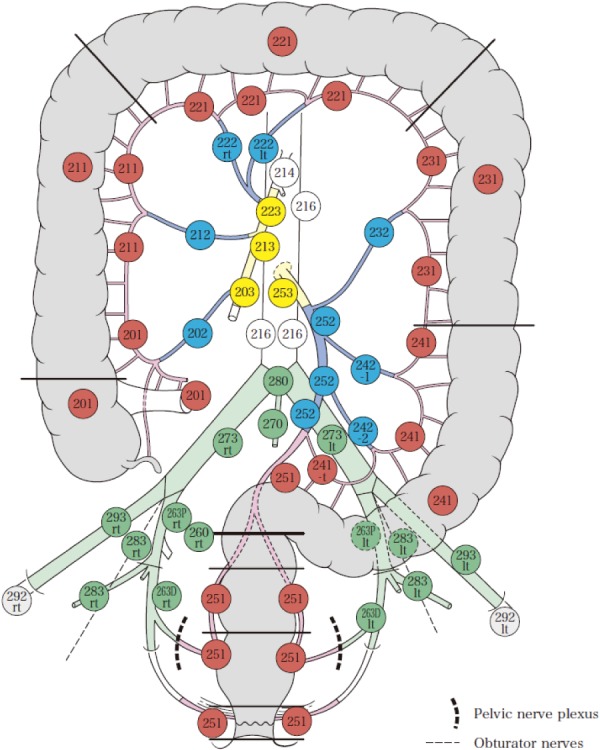

Supplement: Supplementary file 1 — Supplementary file1 (DOCX 156 KB) [file 10151_2025_3187_MOESM1_ESM.docx]
