# Supplementary material for: Safety and feasibility of D3 lymph node dissection in oldest-old patients undergoing colorectal cancer surgery: a multi-institutional, retrospective analysis
Source: Tech Coloproctol. 2025 Jul 19;29(1):146. doi: 10.1007/s10151-025-03187-3 (PMC12276142; doi:10.1007/s10151-025-03187-3)
Supplement: Supplementary file 2 — Supplementary file2 (PDF 11 KB) [file 10151_2025_3187_MOESM2_ESM.pdf]

	pStage II		pStage III	
	D3	non-D3	D3	non-D3
Postoperative complications				
Surgical site infection	2	9	2	5
Pneumonia	3	9	4	0
Urinary tract infection	4	1	5	3
Cerebral infarction	1	1	0	0
Ileus	0	1	1	0
